# The impact of the adoption of a patient rostering model on primary care access and continuity of care in urban family practices in Ontario, Canada

**DOI:** 10.1186/s12875-019-0942-7

**Published:** 2019-04-18

**Authors:** Jatinderpreet Singh, Simone Dahrouge, Michael E. Green

**Affiliations:** 1 Department of Public Health Sciences, Queen’s University, 62 Fifth Field Company Lane, Kingston, ON K7L 3N6 Canada; 2 Department of Family Medicine, University of Ottawa, 600 Peter Morand Crescent, Ottawa, ON K1G 5Z3 Canada; 3 Institute for Clinical Evaluative Sciences, 2075 Bayview Avenue, Toronto, ON M4N 3M5 Canada; 4 Department of Family Medicine, Queen’s University, 220 Bagot St, Kingston, ON K7L 3G2 Canada

**Keywords:** Primary care, Continuity of care, Patient rostering, Primary care access, Fee for service

## Abstract

**Background:**

Greater continuity and access to primary care results in improved patient health, satisfaction, and reduced healthcare costs. Although patient rostering is considered to be a cornerstone of a high performing primary care system and is believed to improve continuity and access, few studies have examined these relationships. This study examined the impact of the adoption of a patient rostering enhanced fee-for-service model (eFFS) on continuity, coordination of specialized care, and access.

**Method:**

A population-based longitudinal study was conducted using health administrative data from urban family practices in Ontario, Canada**.** Family physicians that transitioned from traditional FFS (tFFS) to eFFS between 2004 and 2013 were followed overtime. Physicians providing comprehensive primary care that had at least 4 years of pre-transition and 2 years of post-transition data were eligible. Patients were attributed to physicians on an annual basis by determining the provider that billed the largest dollar amount over a 2 year period. Outcomes of interest were the usual provider of care index (UPC), a referral index (RI) (% of total primary care referrals for a physician’s roster made by the main provider), and emergency department (ED) visits for family practice sensitive conditions (FPSCs). Mixed-effects segmented linear and logistic regressions were used to examine changes in outcomes while controlling for patient and provider contextual factors.

**Results:**

Prior to transitioning, UPC was decreasing at a rate of 0.27%/year (95% CI: -0.34 to − 0.21, *p* < 0.0001). Following the transition, UPC began decreasing by an additional 0.59%/year (95% CI: -0.69 to − 0.49, *p* < 0.0001) relative to the pre-transition rate. RI decreased by an additional 0.34%/year (95% CI: -0.43 to − 0.24, *p* < 0.0001) relative to the pre-transition period, where it had been stable. The transition had minimal impact on FPSC ED visits.

**Conclusion:**

Continuity and coordination of specialized care slightly decreased upon transition from tFFS to eFFS. This is likely due to physicians working in groups and sharing patients following the transition to the eFFS model. Adoption of an enrolment model with after-hours care did not decrease non-urgent ED use, which may reflect the small impact that primary care access has on these types of ED visits.

## Background

Greater access to high quality primary care services results in improved patient health status, increased patient satisfaction, decreased use of hospital resources, and a reduction in overall health system costs [1–4]. In the early 2000s, many nations worldwide initiated primary care reforms in an effort to improve primary care access and overall quality of care [5–8]. Many strategies targeted team structure and changes in remuneration including the introduction of capitation payments, incentive fees, and targeted bonuses [5–8].

A key component of these new models is patient rostering, in part because the new strategies required that patients of physicians be identifiable. Patient rostering (or patient enrolment) is widely considered to be a cornerstone of a high performing primary care system and is currently a key component of family practice models in countries such as Australia, Canada, the Netherlands, Norway, New Zealand, the United Kingdom, and the United States [9]. Patient rostering is a process by which a patient formally registers with a physician (or practice). Through this agreement, the physician (or practice) agrees to provide comprehensive care for their patients, while patients agree to only seek care from their rostered physician unless traveling or in an emergency [10]. Adoption of patient enrolment models also often require physicians to work in groups and collectively provide extended clinical hours (i.e., evenings, weekends, or holidays) for better access [10].

Rostering is thought to help foster accountability, as a given provider has a well-defined patient population for which they are responsible [10]. Furthermore, ongoing access to the same provider over time, which is facilitated through rostering, is perceived to enhance the patient-provider relationship and improve continuity of care [9]. Consistent evidence has shown that increased continuity is associated with improved patient outcomes, increased patient satisfaction, improved coordination of specialist care, reduced hospitalizations and emergency department use, and decreased overall healthcare costs [11–16].

Although it is believed that rostering improves continuity, access, and coordination of specialist care, few studies have examined this relationship. Several cross sectional studies have compared measures of access between different enrolment models [17–20]. For example, Glazier et al. reported that eFFS practices had more after hours care, less emergency room visits, and were caring for patients with higher morbidity [17]. International longitudinal studies have reported the impact of fee for service practices adopting capitation-based enrolment models, however, none attempted to disentangle the potential impact of patient rostering from that of the change in remuneration [9, 21, 22].

In Ontario, Canada’s most populous province, a number of new models of primary care were introduced in the early 2000s [23]. Practices had the choice to transition from a traditional fee-for-service (tFFS) model to a patient enrolment model involving capitation-based payments or they could maintain their fee for service payment structure by transitioning to an enhanced fee for service (eFFS) model that provided increased payments for patients who were rostered [17, 24–26]. Table 1 summarizes the key distinguishing features of each model type [25]. Upon implementation of these new models, a large cohort of practices initially adopted patient rostering alone (ie, the eFFS model) and then later switched to a new model that incorporated both rostering and capitation payments (i.e, Family Health Organization or Family Health Network). The roll out of new primary care models with the sequential adoption of rostering alone (i.e, in the eFFS model) and then capitation payments is an ideal setting to examine the independent impact of rostering on access and continuity.

**Table 1:** Comparison of primary care models in Ontario, Canada

Elements	Fee for service	Enhanced FFS	Blended Capitation
Primary Care Model	Traditional FFS	Family Health Group Comprehensive Care Model	Family Health Network Family Health Organization
Group Size	No minimum	Minimum 3^a^	Minimum 3
Physician Reimbursement	Fee for service	Blended fee for service	Blended capitation
Enrolment	None	Optional	Required
After hours care	No requirement	Required (one 3 h session in evening/weekend per physician per week up to 5 sessions)	Required (one 3 h session in evening/weekend per physician per week up to 5 sessions)
Access bonus (loss of bonus payment for outside primary care use)	No	No	Yes

^a^only 1 physician in a Comprehensive Care Model

We conducted a population level study to examine the impact of the adoption of a patient rostering model (i.e., eFFS) from a tFFS model across Ontario on patient access, continuity of care, and coordination of specialist referrals. This study does not look at the impact of the adoption of capitation-based models. We hypothesized that the adoption of a patient rostering model would improve patient continuity, access, and coordination of specialist referrals.

## Methods

### Study design

We conducted a population-based longitudinal study in order to examine the impact of transitioning from a traditional fee for service (tFFS) model to the enhanced fee for service (eFFS) model. This study looked at outcome measures on a yearly basis before and after practices adopted the eFFS model using health administrative data housed at the Institute for Clinical Evaluative Sciences (ICES) from April 1st, 2000 to March 31st, 2013. This study was approved by the institutional review board at Sunnybrook Health Sciences Centre, Toronto, Canada and the Queen’s University Health Sciences and Affiliated Teaching Hospitals Research Ethics Board, Kingston, Canada (6015466).

### Setting

From 2002 to 2006, the Ontario government introduced various new primary care models with differing physician payment and organizational structures to encourage physicians to shift away from the traditional FFS model (Table 1).

In an attempt to promote patient rostering, the Ontario government introduced two eFFS models – The Family Health Group and the Comprehensive Care Model. In the eFFS model, physicians are strongly encouraged to roster patients, but are not required to offer enrolment to all patients [27]. Physicians in this model receive the majority of their payment through traditional fee-for-service billing, although additional premiums can be obtained for delivering specific preventive care (e.g., pap smear, mammograms, flu shots, colorectal screening) and chronic disease management services (diabetes and chronic heart failure) for rostered patients only [28]. Physicians receive a fee for each patient they roster in their first year ($5 per patient) and a more substantial payment (i.e., $110 - $180 depending on patient age) for enrolling new patients that do not have a family physician (i.e., ‘orphaned’ patients) [29]. Furthermore, physicians receive a small monthly comprehensive care fee per rostered patient and a 10% increase in the amount paid for included core comprehensive FFS codes [29]. Physicians are contractually obligated to provide after-hours care for rostered patients, and those in Family Health Groups must work in a group of three or more physicians.

### Data sources

All health administrative databases required to carry out the analyses in this study were stored at the Institute for Clinical Evaluative Sciences (ICES). These datasets were linked using unique encoded identifiers and analyzed at the Institute for Clinical Evaluative Sciences (ICES) at Queen’s University. Databases at ICES have the advantage of near-complete population coverage (the lowest is OHIP with approximately 94% of visits) [30].

Family physicians that transitioned to eFFS were identified along with their profile using the ICES Corporate Provider Database (CPDB), which captures physician socio-demographic information, their practice model, and location. The Ontario Health Insurance Program database captures all provider billing claims for the provision of care to residents of Ontario who are eligible for insurance coverage. The OHIP database was used to obtain information on referrals to medical specialists.

The Registered Person’s Database captures patient demographic information, including age, sex and postal code for those that are eligible for health insurance coverage in Ontario. The National Ambulatory Care Reporting System (NACRS) provides information on all emergency room encounters.

### Study population

An open cohort of family physicians who transitioned from tFFS to eFFS was created. Physicians in the cohort were followed longitudinally, with their exposure and outcome (see Study outcomes) data being tracked on an annual basis before and after they transitioned to an eFFS model.

Specifically, we identified family physicians who transitioned from tFFS to an eFFS model at any point between April 1st, 2003 and March 31st, 2013 using the ICES Corporate Physician Database, which contains information about their practice model, location, and sociodemographic characteristics. We excluded physicians who were not providing comprehensive family medicine during a given study year (i.e., identified as a specialist in the Corporate Provider Database or billed OHIP for fewer than 8 of the 18 standard primary care fee schedule codes within a given year), had fewer than 100 patients under their care, or had a prolonged absence during a given study year (8 weeks or greater). Furthermore, using the primary practice location for each physician using the Corporate Provider Database, we limited the study to urban physicians as 78% of physicians that transitioned to eFFS were practicing in urban centres and since there are significant contextual differences related to access based on rurality. Also, as mentioned above, many physicians that transitioned to an eFFS model subsequently switched to a capitated model.

For each study year, we identified the group of patients that were under the care of individual study physicians. Patients were included in the study if they had a valid Ontario Health Insurance Plan number and were alive and attributed to a study physician as of March 31st of the fiscal year being examined. Patients were attributed to the physician that billed the largest dollar amount of primary care services for their care over a 2 year period (‘virtual’ attribution method) using the Ontario Health Insurance Program database, which captures all provider billing claims for the provision of care to residents of Ontario who are eligible for insurance coverage [31]. Since the Client Agency Program Enrolment (CAPE) dataset only identifies rostered patients for the eFFS practices in this study and not those in the tFFS model, we used the ‘virtual’ attribution method to create provider rosters both pre- and post-transition, despite the fact that the CAPE database tracks official patient rostering for eFFS practices. This method has been used in previous studies and is the accepted reporting method of the Ministry of Health and Long Term Care of Ontario [31]. This was done to avoid differential misclassification that would have resulted from using a different attribution method for patients before and after the transition to eFFS. Previous work done by our group has shown that the percentage agreement between the virtual rostering method and the CAPE database is greater than 85% (see limitations for further discussion) [32].

In addition, patients were excluded if they did not have a primary care visit to their family physician for two consecutive years (i.e., during the year of interest and the year prior). Eligible patients were subsequently linked to the Registered Person’s Database, which captures patient demographic information, including age, sex and postal code for those that are eligible for health insurance coverage in Ontario.

To ensure the pre-transition phase was adequately captured, we then excluded physicians with less than 4 years of pre-transition data. Similarly, physicians with less than 2 years of post-transition data were excluded. This occurred because the physician transitioned within that period from eFFS to another model, or because they moved from the province. Follow up of physicians was discontinued if they transferred out of the eFFS model into another model (eg, capitation model) type (i.e, if a physician subsequently switched to capitation, only data collected during years that they were in tFFS and eFFS were used in the analysis).

### Study outcomes

We assessed measures of continuity of care, coordination of specialist care, and primary care access.

Relational continuity of care was assessed using the Usual Provider of Care Index (UPC). UPC is a patient level outcome that looks at the percentage of primary care visits to the main provider relative to all primary care visits (i.e., high UPC = better continuity) over a 2 year period (i.e., fiscal year of interest and the year prior) [33]. Patients with less than three visits over the 2 year span were excluded from the analysis, as data for these patients tend to cluster around 0, 50, and 100%, which has been shown to impact the reliability of this measure [34]. The UPC index is a validated measure that is commonly used to assess continuity [35].

In order to assess coordination of specialist care, we developed a referral index (RI). RI is a physician level measure that represents the percentage of total primary care referrals for a physician’s roster made by the main provider (i.e, as opposed to referrals made by walk-in physicians or other family physicians). Since diagnostic radiology makes up a large percentage of all referrals and does not represent a traditional referral per se, they were excluded from this metric. Also, referrals to allied health professionals was not assessed in this outcome.

Lastly, access was assessed using non-urgent emergency department (ED) visits. Non-urgent ED visits is a commonly used proxy for primary care access [25, 36, 37]. Specifically, this study looked at the number of ED visits (Source: NACRS) for family practice sensitive conditions (FPSCs) on the patient level. These ED visits are for health conditions that are less urgent and have less than a 1% chance of an inpatient visit, and thus, represent conditions that would more appropriately be handled in a primary care setting [38]. Examples of FPSCs include conditions such as conjunctivitis, otitis media, acute pharyngitis, sinusitis, and acute upper respiratory tract infection. This measure was established by the Health Quality Council of Alberta, and has been used as a proxy measure for primary care access by organizations such as the Canadian Institute of Health Information. Since there was a coding change in NACRS in 2002 that would have impacted this outcome, we only looked at data for this outcome from 2003 to 2013. Since the percentage of individuals across Ontario that have a FPSC ED visit is quite low, and the majority that do, only have a single visit, this outcome measure was treated as a dichotomous outcome.

### Analysis

We used mixed-effects segmented linear and logistic regression models to examine changes in outcomes while controlling for patient and provider contextual factors. This approach divides the data into pre- and post-intervention periods, determining separate intercepts and slopes for each time period [39]. Statistical tests were used to compare the intercepts and slopes of each line to see if the transition to eFFS resulted in a change in outcome measures that was significantly greater than any underlying secular trend. All models accounted for the clustering of patients to providers using a generalized mixed effects model. The intercept, time (measured as a continuous variable in years), type of care model, and time after transition (measured as a continuous variable in years) were all assigned as random effects in all models to deal with the heterogeneity within the data across physicians.

A multivariate logistic regression model was used to assess FPSC ED visits, while a multivariate linear regression was used for continuous measures (i.e., UPC, RI). All models adjusted for both patient (age, sex, socioeconomic status via neighbourhood income quintile, urban/rural residence, case mix) and provider (sex, years since graduation, foreign medical training, and total number of patients under the care of each physician (i.e., panel size)) level contextual factors as they have all been shown to impact access, continuity, and specialist referrals in previous studies [19, 40–43]. Since the above models assume linear trends over time, descriptive linear plots were constructed (for the overall population and for cohorts belonging to each individual transition year) in order to ensure the data followed a linear trend.

Furthermore, previous studies have shown that a portion of patients (approximately 15%) opt not to formally roster with their physicians after they transition to enrollment models [32, 44]. Since the intent of the Ontario Government was to have all patients rostered, these patients were kept in the main analysis and assessed in the eFFS group even though they were not formally rostered.

A secondary analysis was done to compare the impact of the transition to eFFS on early versus late adopters of the new model, as previous studies have demonstrated differences between both groups [28]. An interaction term for early adoption (i.e, early_adoption = 1 for physicians that transitioned between 2004 to 2006, early_adoption = 0 for those that transitioned from 2007 to 2011) was added to each model to examine if there was a differential impact on early versus late adopters of the eFFS model. Plots of these analyses were created by setting patient and provider factors to their mean and mode values. All analyses were conducted using SAS, Version 9.3, SAS Institute Inc.

## Results

There were 3291 physicians included in this study. The majority of these physicians transitioned between 2004 and 2006 (*n* = 2832), were male (63.5%), and were Canadian trained (76.0%). They had an average panel size of 1478 (Standard deviation (SD) = 645) and had been in practice 24.7 (SD 9.5) years during their transition year. The patient profile of these physicians during the transition year is shown in Table 2.

**Table 2:** Physician and Patient Characteristics during the year of transition

Characteristic	N	Percentage (%)
Physicians		
Sex		
Male	2088	63.5
Female	1203	36.5
Canadian Trained	2502	76.0
Panel size		
< 500	129	3.9
500–999	676	20.5
1000–1999	1834	55.7
2000–2999	580	17.6
> 3000	72	2.2
Years since Grad (mean, SD)	24.7 (9.5)	
Patients		
Sex		
Male	1,655,749	43.6
Female	2,143,143	56.4
Age (mean, SD)	41.4 (22.1)	
</=19	750,427	19.8
20–39	956,271	25.2
40–59	1,264,563	33.3
60–79	677,578	17.8
>/=80	150,053	4.0
Rurality		
Urban	3,528,411	92.9
Sub-urban	228,313	6.0
Rural	34,190	1.0
Missing	7978	0.02
Income Quintile^a^		
1	726,353	19.1
2	750,767	19.8
3	753,366	19.8
4	773,195	20.4
5	788,390	20.8
Missing	6821	0.2
Adjusted Clinical Group (ACG)^b^		
0	47,988	1.3
1–4	1,395,673	36.7
5–9	1,901,853	50.1
10+	453,378	11.9

^a^income quintile represents the rank of the patient’s total household income based on the aggregate census data derived from postal code. The first quintile represents the highest incomes

^b^Adjusted Clinical Groups (ACG) quantifies morbidity by grouping patients based on age and gender and all medical diagnoses in a given year. Those in group three represent represents those with the greatest morbidity

**Fig. 1:**
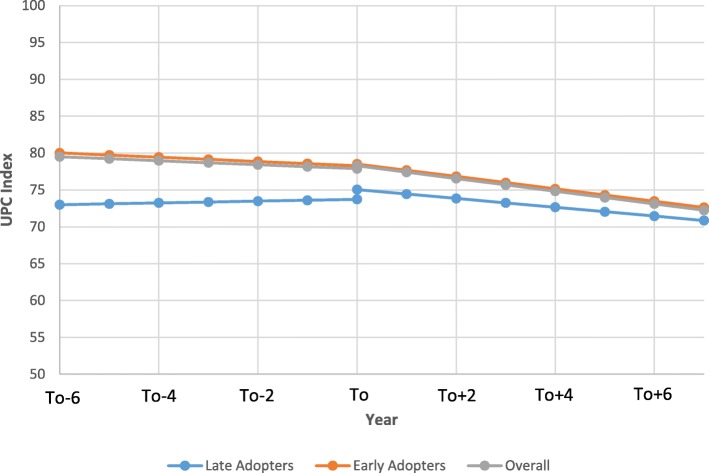
Comparison of the impact of the transition from tFFS to eFFS on Usual Provider of Care (UPC) index between early, late, and the overall population (To = year of transition). (note: for female patient that is 41, 5th income quintile, 2nd quintile for acg, with a male Canadian trained physician with a panel size between 1000 and 1999 who graduated 25 years ago)

Table 3 presents the results of the regressions accounting for the clustering structure of the data only (i.e., unadjusted model) and adjusted models examining the change in UPC index following the transition from tFFS to eFFS. After adjustment for provider and patient level factors, the UPC index showed a slight trend towards decreasing continuity over time (0.27%/year (95% confidence interval (CI): − 0.34 to − 0.21)) in the years prior to the transition. Following the transition, this trend intensified, as the UPC began decreasing by an additional 0.59%/year (95% CI: -0.69 to − 0.49) (*p* < 0.0001) relative to the pre-transition baseline rate. There was minimal change in UPC during the year of transition (0.39, 95%CI 0.23 to 0.55, *p* < 0.0001). A physician having transitioned in 2004 would have had a drop of 8.6% in UPC over the following 10 years. Figure 1 presents the adjusted segmented regression model for the UPC for the overall model and also for the early and late adopters. Early adopters had a 7.02% (95% CI: 5.98 to 8.06, *p* < 0.0001) higher baseline UPC than the late adopters. Similar to the overall UPC analysis, the post-transition UPC rate decreased relative to the pre-transition slope for both the early (− 0.724%/year, 95%CI: -0.990 to − 0.458) and late adopters (− 0.570%/year, 95% CI: -1.12 to − 0.018). UPC index was higher for providers that were male, Canadian trained, had larger panel sizes, and more years since graduation and for patients that were male, older, and healthier.

**Table 3:** Segmented linear regression results examining impact of transition from tFFS to eFFS on UPC index

Parameter	Unadjusted Model			Adjusted Model		
	Estimate	95% CI	*P*-Value	Estimate	95% CI	*P*-Value
Intercept (baseline UPC)	75.9	75.5 to 76.3	< 0.0001	57.2	56.3 to 58.1	< 0.0001
Pre-intervention slope (secular trend, per year)	0.35	0.30 to 0.41	< 0.0001	−0.27	− 0.34 to − 0.21	< 0.0001
Change in intercept (immediate impact)	0.42	0.45 to 0.58	< 0.0001	0.39	0.23 to 0.55	< 0.0001
Change in slope (gradual effect, per year)	−0.72	− 0.82 to − 0.61	< 0.0001	− 0.59	−0.69 to − 0.49	< 0.0001
Female physician	−1.05			−1.05	−1.80 to − 0.29	0.007
Physician panel size						
< 500				0		
500–999				4.10	3.97 to 4.23	< 0.0001
1000–1999				6.83	6.68 to 7.37	< 0.0001
2000–2999				7.52	7.37 to 7.67	< 0.0001
> 3000				8.16	7.99 to 8.34	< 0.0001
Foreign Trained				−2.59	−3.47 to −1.70	< 0.0001
Years since graduation				0.43	0.39 to 0.47	< 0.0001
Patient age				0.30	0.30 to 0.30	< 0.0001
Female patient				−0.96	−0.97 to − 0.94	< 0.0001
Adjusted Clinical Group (ACG)^b^						
0				0		
1–4				−2.60	−2.66 to − 2.55	< 0.0001
5–9				−6.10	−6.16 to −6.05	< 0.0001
10+				−9.18	−9.24 to −9.12	< 0.0001
Income Quintile^a^						
1				0		
2				−0.002	−0.021 to 0.018	0.88
3				−0.29	−0.31 to − 0.27	< 0.0001
4				− 0.36	− 0.38 to − 0.34	< 0.0001
5				− 0.37	− 0.39 to − 0.35	< 0.0001
Patient rurality						
Urban				0		
Suburban				− 0.26	− 0.29 to − 0.23	< 0.0001
Rural				−1.41	−1.48 to −1.35	< 0.0001

^a^income quintile represents the rank of the patient’s total household income based on the aggregate census data derived from postal code. The first quintile represents the highest incomes

^b^Adjusted Clinical Groups (ACG) quantifies morbidity by grouping patients based on age and gender and all medical diagnoses in a given year. Those in group three represent represents those with the greatest morbidity

**Fig. 2:**
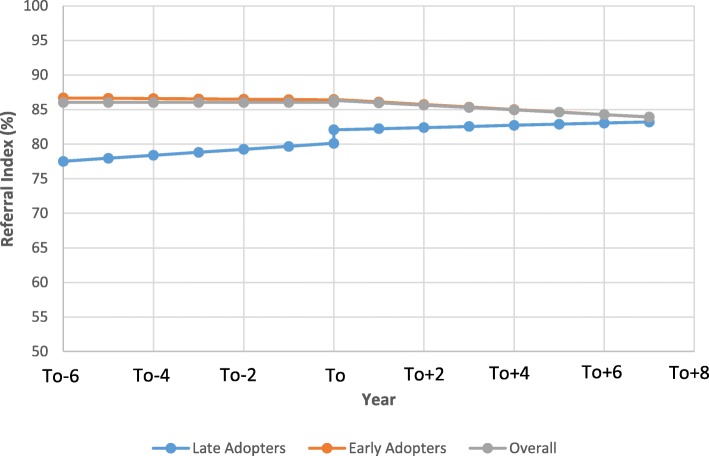
Comparison of the impact of the transition from tFFS to eFFS on Referral Index (RI) between early, late, and the overall population (To = year of transition). (note: for male Canadian trained physician with a panel size between 1000 and 1999 who graduated 25 years ago)

Trends observed for RI were very similar to those observed for the UPC index (Table 4). After adjustment for provider level factors, the RI slope was negligible over time prior to transition. Following the transition, RI decreased by 0.34%/year (95% CI: -0.43 to − 0.24, *p* < 0.0001). Figure 2 compares the impact of the transition from tFFS to eFFS between the early and late adopters. The early adopters had a 9.17% (95% CI: 7.91 to 10.43, p < 0.0001) higher baseline RI than the late adopters. Following the transition, the RI decreased relative to the pre-transition slope in a manner that was similar between the late (− 0.273, 95% CI: -0.533 to − 0.013) and early adopters (− 0.318, 95% CI: -0.857 to 0.220). RI was higher for providers that were male, Canadian trained, had larger panel sizes, and more years since graduation.

**Table 4:** Segmented linear regression results examining impact of transition from tFFS to eFFS on RI

Parameter	Unadjusted Model			Adjusted Model		
	Estimate	95% CI	*P*-Value	Estimate	95% CI	*P*-Value
Intercept (baseline RI)	81.7	81.2 to 82.3	< 0.0001	60.6	59.4 to 61.8	< 0.0001
Pre-intervention slope (secular trend, per year)	0.52	0.45 to 0.59	< 0.0001	−0.0014	− 0.078 to 0.075	0.97
Change in intercept (immediate impact)	0.41	0.21 to 0.62	0.0001	0.29	0.04 to 0.54	0.02
Change in slope (gradual effect, per year)	−0.43	−0.50 to − 0.36	< 0.0001	− 0.34	−0.43 to − 0.24	< 0.0001
Female physician				4.22	3.29 to 5.15	< 0.0001
Physician panel size						
< 500				0		
500–999				9.27	8.81 to 9.73	< 0.0001
1000–1999				12.6	12.1 to 13.1	< 0.0001
2000–2999				13.4	12.8 to 14.0	< 0.0001
> 3000				14.1	13.1 to 15.1	< 0.0001
Foreign Trained				−6.4	−7.5 to −5.4	< 0.0001
Years since graduation				0.52	0.47 to 0.57	< 0.0001

**Fig. 3:**
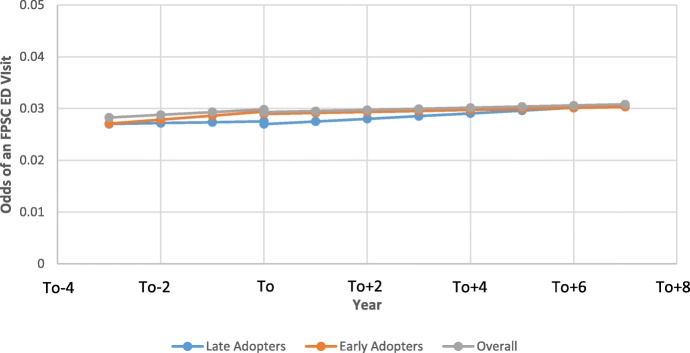
Comparison of the impact of the transition from tFFS to eFFS on the odds of a family practice sensitive condition emergency department (FPSC-ED) visits between early, late, and the overall population (To = year of transition). (note: for female patient that is 41, 5th income quintile, 2nd quintile for acg, with a male Canadian trained physician with a panel size between 1000 and 1999 who graduated 25 years ago)

Prior to transitioning, the odds of FPSC ED visits was increasing by 1.02 (95% CI: 1.015 to 1.021) fold per year (Table 5). Following the transition, the odds of a FPSC ED visit continued increasing, but at a slightly slower rate compared to the pre-transition rate (OR = 1.007 fold per year, 95% CI: 1.001 to 1.013, *p* < 0.001). Figure 3 presents the results of the segmented regression for the entire population and for both the early and late adopters. As can be seen from Fig. 3, the level of FPSC emergency department visits remained fairly stable following the transition.

**Table 5:** Segmented logistic regression results examining impact of transition from tFFS to eFFS on FPSC ED Visits

Parameter	Unadjusted Model			Adjusted Model		
	Estimate^a^	95% CI	*P*-Value	Estimate	95% CI	*P*-Value
Intercept (baseline ED)	−3.51	−3.53 to −3.50	< 0.0001	−3.28	−3.33 to −3.23	< 0.0001
Pre-intervention slope (secular trend, per year)	0.015	0.013 to 0.019	< 0.0001	0.018	0.015 to 0.021	< 0.0001
Change in intercept (immediate impact)	−0.011	−0.014 to −0.0070	0.0098	−0.010	−0.018 to − 0.0020	0.0128
Change in slope (gradual effect, per year)	−0.010	− 0.014 to − 0.0068	< 0.0001	−0.011	− 0.014 to − 0.0080	< 0.0001
Female physician				−0.041	− 0.089 to − 0.022	0.0123
Physician panel size						
< 500				0		
500–999				−0.021	−0.055 to − 0.060	0.015
1000–1999				− 0.030	−0.067 to − 0.015	0.0018
2000–2999				− 0.070	−0.098 to − 0.042	< 0.0001
> 3000				− 0.074	− 0.110 to − 0.039	< 0.0001
Foreign Trained				− 0.23	−0.27 to − 0.19	< 0.0001
Years since graduation				0.0058	0.0040 to 0.0080	< 0.0001
Patient age				−0.021	−0.022 to − 0.021	< 0.0001
Female patient				− 0.047	− 0.051 to − 0.044	< 0.0001
Adjusted Clinical Group (ACG)^c^						
0				0		
1–4				0.20	0.19 to 0.21	< 0.0001
5–9				0.79	0.77 to 0.80	< 0.0001
10+				1.53	1.52 to 1.56	< 0.0001
Income Quintile^b^						
1				0		
2				−0.12	−0.12 to 0.11	0.88
3				−0.194	−0.200 to − 0.189	< 0.0001
4				− 0.256	− 0.261 to − 0.251	< 0.0001
5				− 0.333	−0.338 to − 0.327	< 0.0001
Patient rurality						
Urban				0		
Suburban				0.65	0.64 to 0.65	< 0.0001
Rural				1.31	1.29 to 1.31	< 0.0001

^a^Estimates represent the log odds of an FPSC ED visit

^b^income quintile represents the rank of the patient’s total household income based on the aggregate census data derived from postal code. The first quintile represents the highest incomes

^c^Adjusted Clinical Groups (ACG) quantifies morbidity by grouping patients based on age and gender and all medical diagnoses in a given year. Those in group three represent represents those with the greatest morbidity

## Discussion

This comprehensive population-based analysis showed that measures of continuity of care and coordination of specialized care experienced a small decrease upon transition from tFFS to a patient rostering eFFS model. The transition had statistically significant change on FPSC emergency department visits, however, these changes were of minimal clinical significance. Although several previous cross-sectional studies have compared measures of access and continuity between different primary care models, this is the first longitudinal study to examine the impact of the adoption of a rostering model on measures of continuity, coordination of specialty care, and access [17, 20].

Following the transition to eFFS, the UPC began decreasing by an additional 0.59%/year (95% CI: -0.69 to − 0.49, *p* < 0.0001) compared to the pre-transition rate. This is likely due in part to the change in group structure experienced by physicians once they transitioned to an eFFS model. As mentioned above, physicians within this eFFS model were required to work in groups (i.e., a minimum of 3 physicians per group) and share after-hours coverage, which required physicians to see patients rostered under other group members. Thus, in cases where a patient may have previously waited until the next day to see their regular provider in a tFFS model, the eFFS model allowed them to see another provider in the after-hours clinic which would allow for more timely care but decreases the UPC index. Since data about group practices was not available for tFFS practices (see limitations), we were not able to do an analysis for group practice level continuity. We looked at group level continuity post-transition and found that during the year 2013, the group level UPC was 5.5% higher than the provider level continuity for that year. This difference makes up for the decreased UPC observed in Fig. 1 following the transition to eFFS (i.e, 4.2% decrease from 2006 to 2013).

Continuity of care has been shown to be associated with decreased hospitalizations and emergency department use, increased patient satisfaction, and improved patient outcomes [11–13, 15, 45]. For example, a recent 17-year prospective cohort study in Amsterdam showed that those with low versus high provider level continuity had an increased risk of mortality [15]. Also, previous studies have shown that a 10% increases in UPC correlates with a 2% reduction in preventable hospitalizations and a 6–8% decrease in emergency room utilization [46, 47]. The majority of benefits of continuity studied to date have focussed on provider level continuity with much less evidence demonstrating the benefits of practice level continuity [48]. Of those studies that have examined both levels of continuity, current evidence suggests that those having a usual provider of care as opposed to simply having a regular practice site results in improved preventive care [12], diabetes care [49], reduced medication duplication [50], and increased patient satisfaction [51]. The above demonstrates the importance of maintaining an ongoing relationship with the same provider over time.

Similarly, there was a small decrease in RI following the transition to the eFFS model. This is not surprising given the observed decrease in UPC. As patients see more physicians, one would expect that there is an increased likelihood that patients receive specialist referrals from multiple providers. Previous evidence has shown that non-assigned primary care providers have an increased likelihood of referring a patient to a specialist relative to their primary family physician [52]. The link between continuity of care and coordination of specialist care has been demonstrated in the past. For example, a study by O’Malley et al. (2009) found that family physicians whose patients had higher continuity of care were better informed about recent specialist visits and more consistently discussed these visits with their patients than those with lower continuity [53].

Although the transition to eFFS increased the number of patients in Ontario with a regular family doctor and offered after hours care, this minimally decreased the utilization of ED for less urgent presentations [26]. For example, using data for all of Ontario in 2013/2014, the change observed in FPSC ED visits per year due to the transition would equate to 6 fewer visits per 10,000 Ontarians. Although previous cross sectional studies have demonstrated that patients in eFFS models visit emergency rooms less than other models in Ontario [17], this is more likely related to the characteristics of the providers (and their patients) who self-selected into an eFFS practice, as opposed to the model itself. The findings in this study demonstrating little impact on FPSC ED visits is in line with other Ontario-based studies [24]. Although adoption of the eFFS model was meant to allow patients to receive more timely access, evidence from recent studies highlights that there was no improvement in a patients ability to obtain same-day access [26, 27]. This appears to be due in part to the implementation and monitoring of after-hours care in the province. An evaluation conducted by the Auditor General of Ontario in 2011 highlighted several concerns with the manner in which physicians in enrolment models were providing after-hours care [27]. Group practices were obligated to provide at least one three hour block of after-hours care per week for each physician to a maximum of five blocks within a week [27]. However, 53% of eFFS had more than five members, meaning that larger groups were not necessarily providing proportionately greater after hours care. Also, the Auditor General highlighted that ongoing monitoring to ensure practices were meeting these obligations needed to be improved as only 74% of the eFFS practices were found to be providing after-hours care in accordance with their contractual requirements. Lastly, even though most eFFS groups were operating out of multiple practices sites, after-hours services were only required at a single site which may not have been convenient for all enrolled patents in the group [27].

Furthermore, although there is evidence that improved access to primary care reduces less urgent emergency department utilization, there is a growing body of evidence which demonstrates that there are other important factors involved including the complex nature of individual decision making on when the emergency department is needed [54, 55]. For example, a recent Ontario-based study done by Green et al. patients decision to visit the emergency department was primarily related to the fact that they felt that it was medically necessary and less to do with difficulty accessing their primary care physician [54]. Thus, although the eFFS model offered after hours care and physicians within these models were working more often, this did not impact FPSC ED visits.

Lastly, there were significant differences between the early and late adopters of the eFFS model, particularly with respect to UPC and RI. In both cases, the early adopters had significantly higher baseline performance (i.e, UPC and RI) relative to the late adopters. Also, immediately following the transition, the late adopters saw a significant increase in their UPC and RI levels (Figs. 1 and 2), whereas this was seen to a lesser extent in the early adopters. The differences between both groups in UPC and RI diminished during the duration of the study timeframe. This is likely due to the fact that those providers whose practices were more established and had to change their practices to a lesser extent to transition to eFFS were more likely to be in the early adopter group [28]. Since the late adopters had lower baseline levels of UPC and RI, they had more opportunity for growth and thus, they had a greater increase in performance immediately following the transition [28]. That being said, it appears that as newly formed eFFS groups got more settled and comfortable within their new models, they likely began sharing patients amongst one another which played a role in slope for both the UPC and RI measures decreasing in subsequent years following the transition. Similar findings have been seen in past studies as early adopters tend to have higher baseline performance [28] . For example, a study conducted by Kantarvic et al. looked at changes in practice patterns following physician transitions from tFFS to eFFS in Ontario. Overall, this study found that physicians in Ontario were more productive after transitioning from tFFS to eFFS as measured by number of services delivered, number of visits, and also distinct patients seen. This study also found that the early adopters of the eFFS model in Ontario had higher baseline productivity, with the transition having a greater impact on the later adopters [28]. Also, the study by Kantarvic et al. provided evidence that indicated that practices likely began altering their practices the year prior to transitioning, which may explain the sudden jump in UPC and RI seen for the late adopters in this study.

### Strengths and limitations

This study has a number of important strengths. This study uses administrative databases that has near complete population coverage (the lowest is the OHIP database with 94% completion) which minimizes participation bias and ensures that this study is adequately powered to detect clinically relevant changes in outcomes [30]. Collectively, the databases housed at ICES provide a wealth of information on the patient, provider, and practice level, which allowed for appropriate adjustment within regression models. Furthermore, this broad spectrum of data allowed us to look at diverse measures of access to provide a comprehensive assessment of the impact of the adoption of the eFFS enrolment model on access. This was a longitudinal study that utilized data over a 13 year period, and thus, allows for insights into potential causation. Lastly, the staggered nature of the adoption of eFFS model and then capitation models in Ontario facilitated the ability of this study to look at the impact of the adoption of patient rostering in isolation of a change to a capitation payment approach.

There are also several limitations. This study utilized a quasi-experimental design that does not adequately control for temporal changes as can be done in a well-designed randomized control trial. That being said, the offset in the transition to eFFS allowed us to control for potential temporal changes as late adopters acted as temporal controls for early adopters (i.e, late adopters transitioned from 2007 to 2011, and thus remained in the tFFS model and acted as a control for the early adopters between 2004 and 2006). That being said, this approach assumes that early and late adopters have similar underlying secular trends and would experience sudden changes the same way, which is not always the case, and thus, we are unable to definitely conclude causation of any of these findings. This is a common limitation of studies examining policy changes in which RCTs are not feasible.

Furthermore, the statistical model used in this study assumes a linear trend over time. We constructed descriptive plots for the overall population and for each individual cohort of physicians based on transition year and all plots were predominantly linear. Also, the overall model assumes that the impact of the transition to eFFS was the same across providers that transitioned at different times. A secondary analysis compared early versus late adopters and showed that although there were baseline differences between the two groups, the impact of the transition to eFFS resulted in differences between early versus late adopters that were of only minimal clinical significance.

As was mentioned above, one potential reason for the observed downward trend in the physician level UPC measure was that physicians where sharing more care responsibilities with their team members. Thus, it would have been valuable to do an analysis of a practice level UPC to see if rostering improved overall continuity within the practice. Although information on practice groups is readily available through the Client Agency Program Enrolment (CAPE) database for those in eFFS models, there is no validated approach to identify potential groups in tFFS practices. Although it is likely that some physicians that shared practice space also saw each other’s patients in tFFS practices, this was not a formalized grouping prior to the switch to an enrolment model. As such, we were limited in our ability to assess the impact of the adoption of a rostering model on practice level continuity and would have made it difficult to interpret other measures of continuity that are better reflections of team-based care (eg, continuity of care index), visit entropy [56]). That being said, as mentioned above, there is evidence that provider level continuity is more important for patient satisfaction and outcomes [12, 49, 51], making these findings highly relevant.

Furthermore, the current method used to identify patients in tFFS practices and non-rostered patients in the eFFS practices is the ‘Virtual Rostering’ method. This method has been used in previous studies and is the accepted reporting method of the MOHLTC [57, 58]. We anticipate that utilization of this approach resulted in random error in attributing patients to their family physician. Previous work from our group has shown that this misattribution can be as high as 15% [32]. Although the Client Agency Program Enrolment (CAPE) database identifies all patients that are rostered to a given physician, we used the ‘Virtual rostering’ for all patients in this study irrespective of their enrolment status or model in order to avoid any differential misclassification across patients before and after they transitioned to the eFFS model.

Also, since the virtual rostering method relies on a patient’s primary care billings over a 2 year period, those patients with no visits over a specific 2 year period were excluded from the analysis during that specific time frame. Similarly, since the UPC index tends to be a skewed measure, those patients with less than 3 visits over a 2 year period were not included in the assessment of this particular measure, as the values of UPC tend to cluster around 0, 50%, and 100. Thus, the results of this study do not reflect the experiences of very infrequent users of the primary care system. That being said, pre?A3B2 show $132#?>vious work done through ICES has shown that 90% of enrolled patients have at least a single visit to a primary care physician over the 2 year timeframe used with the virtual rostering method (Alexander Kopp, ICES, January 19, 2019).

## Conclusions

This study examined the impact of transitioning from a tFFS model to an eFFS patient rostering model on access, continuity, and coordination of specialist care. A long held belief has been that patient rostering models that offer after-hours care help improve patient-provider continuity and have the potential to reduce non-urgent emergency department visits that could be managed in a primary care setting. The results from this study demonstrated that the adoption of an eFFS patient rostering model resulted in decreased provider level continuity and coordination of specialist care, and had little impact on FPSC ED visits. As physicians began sharing patients with other eFFS group members, this likely increased timely access to care but decreased provider level continuity of care. In Ontario, over $1 billion per year was spent on the adoption of these new models, and as such, it is of importance to clearly understand the impact that these new models had on patient care. Although this model has been shown in previous studies to enhance physician productivity, future studies should examine what impact the observed changes in continuity and coordination of specialist referrals has had on patient outcomes. Also, other models in Ontario involve capitation payments and access bonuses, and as such, it would be of interest to see if these features impacted access and continuity in a manner that was different than the model investigated in this study.

## Abbreviations

ACG: Adjusted Clinical Groups
CAPE: Client Agency Program Enrolment
CPDB: Corporate Provider Database
ED: Emergency department
eFFS: Enhanced fee-for-service
FPSC: Family Practice Sensitive Condition
ICES: Institute for Clinical Evaluative Sciences
NACRS: National Ambulatory Care Reporting System
OHIP: Ontario Health Insurance Plan
RI: Referral Index
tFFS: Traditional fee-for-service
UPC: Usual Provider of Care
